# Prognostic Value of the Tricuspid Regurgitation Impact on Outcomes (TRIO) Score in Patients Undergoing Transcatheter Aortic Valve Implantation

**DOI:** 10.31083/RCM26504

**Published:** 2025-01-21

**Authors:** Jizhong Wang, Yuanwei Chen, Xuxing Zhang, Songyuan Luo, Jie Li, Fang Pei, Jianfang Luo

**Affiliations:** ^1^Guangdong Cardiovascular Institute, Guangdong Provincial People's Hospital Ganzhou Hospital, Guangdong Academy of Medical Sciences, 341000 Ganzhou, Jiangxi, China; ^2^Department of Cardiology, Guangdong Provincial People's Hospital (Guangdong Academy of Medical Sciences), Southern Medical University, 510080 Guangzhou, Guangdong, China

**Keywords:** transcatheter aortic valve implantation, TRIO score, mortality, major adverse cardiovascular events

## Abstract

**Background:**

Prognosis assessments for transcatheter aortic valve implantation (TAVI) patients remain challenging, particularly as the indications for TAVI expand to lower-risk patients. This study assessed the prognostic value of the tricuspid regurgitation impact on outcomes (TRIO) score in patients after TAVI.

**Methods:**

This single-center study included 530 consecutive patients who underwent TAVI. Patients with a TRIO score >4 were compared to those with a score ≤4. The primary outcome was all-cause mortality, while secondary outcomes included complications defined by the Valve Academic Research Consortium 2 (VARC-2) criteria and major adverse cardiovascular events (MACEs), including mortality, stroke, and heart failure rehospitalization.

**Results:**

Over a mean follow-up period of 22 months, patients with a TRIO score >4 had significantly higher rates of mortality (11.5% vs. 3.1%, *p* < 0.001) and MACEs (14.9% vs. 3.6%, *p* < 0.001). Multivariable Cox regression analysis identified a TRIO score >4 as an independent risk factor for all-cause mortality (hazard ratio (HR): 2.41, 95% confidence interval (CI): 1.08–5.37, *p* = 0.032) and MACEs (HR: 2.78, 95% CI: 1.34–5.75, *p* = 0.006). Patients with a higher TRIO score also had significantly higher rates of stroke (3.1% vs. 0.5%, *p* = 0.028), acute kidney injury (10.1% vs. 4.3%, *p* = 0.011), and MACEs (14.9% vs. 3.6%, *p* < 0.001) within 30 days after TAVI.

**Conclusions:**

The TRIO score was associated with all-cause mortality and MACEs in patients after a TAVI. The TRIO score could serve as a convenient tool for risk stratification in clinical practice, aiding in identifying high-risk patients.

## 1. Introduction

Over the past decade, the indications for transcatheter aortic valve implantation (TAVI) have expanded from targeting high-risk patients to encompassing the entire risk spectrum for individuals with aortic stenosis (AS) [1]. Following recent extensions to younger and low-risk patients with a longer life expectancy [2], selecting appropriate TAVI candidates and accurately stratifying their risk remains challenging. Traditional risk assessment tools, such as the Society for Thoracic Surgery Predictive Risk of Mortality [3] and the logistic European System for Cardiac Operative Risk Evaluation [4], were originally developed to assess the risks associated with conventional cardiac surgery rather than a TAVI. Subsequently, concise and accurate tools that can specifically discriminate prognosis following TAVI remain limited.

Concomitant tricuspid regurgitation is common in patients undergoing a TAVI and has been associated with poorer outcomes [5, 6, 7]. The tricuspid regurgitation impact on outcomes (TRIO) score was developed to assess mortality risk in patients with tricuspid regurgitation, incorporating variables such as demographics, laboratory parameters, and echocardiographic findings [8]. However, utilizing the TRIO score in risk stratification for patients undergoing TAVI remains uncertain.

This study evaluated the prognostic value of the TRIO score in patients after a TAVI. We hypothesized that the TRIO score could effectively identify high-risk patients with a greater likelihood of adverse outcomes following a TAVI.

## 2. Methods

### 2.1 Study Design

We conducted a retrospective analysis of patients who underwent a TAVI between January 2016 and December 2022 at Guangdong Cardiovascular Institute, Guangdong Provincial People’s Hospital, China. The current study enrolled patients who underwent TAVI between January 2016 and December 2022. No exclusion criteria were applied. A TAVI was performed in patients with symptomatic severe AS using a standard approach. Patients received either self-expandable or balloon-expandable valves via transfemoral, trans-carotid, trans-subclavian, or transapical routes under general or local anesthesia, as determined by individual heart teams based on multidetector computed tomography. All patients were followed up with at 1 month, 6 months, and annually until July 2023. This study was approved by the Research Ethics Committee of Guangdong Provincial People’s Hospital (No. GDREC2019384H), and informed consent was obtained from all participants.

### 2.2 Clinical Variables

Demographic, laboratory, and echocardiographic data were collected before the TAVI. The TRIO score comprises eight weighted variables [8]: age (<70 years, 0 points; 70–79 years, 1 point; ≥80 years, 2 points), sex (female, 0 points; male, 1 point), renal function (creatinine <2 mg/dL, 0 points; creatinine ≥2 mg/dL, 2 points), congestive heart failure (no, 0 points; yes, 2 points), lung disease (no, 0 points; yes, 2 points), aspartate aminotransferase (<40 U/L, 0 points; ≥40 U/L, 1 point), heart rate (<90 beats/min, 0 points; ≥90 beats/min, 1 point), and severe tricuspid regurgitation (no, 0 points; yes, 1 point) [8]. The study population was categorized into low (≤ median) and high (> median) TRIO score groups.

### 2.3 Clinical Outcomes

The primary endpoint was all-cause mortality at the latest follow-up after the index procedure. Secondary endpoints included complications at 30 days, as defined by the Valve Academic Research Consortium-3 criteria [9]. Major adverse cardiovascular events (MACEs) were defined as a composite of mortality, stroke, and heart failure rehospitalization at 30 days and the latest follow-up.

### 2.4 Statistical Analysis

Continuous variables are presented as the mean ± standard deviation or median with interquartile range (IQR), depending on variable distribution, and compared using Student’s *t*-test or the Mann–Whitney U test, respectively. Categorical variables are expressed as percentages and compared using the chi-squared test or Fisher’s exact test, as appropriate. Multiple imputations were used for missing values.

Univariable and multivariable logistic regression models were utilized to evaluate the association between TRIO scores and 30-day outcomes, with odds ratios (ORs) and 95% confidence interval (CI) reported. Survival curves were constructed using the Kaplan–Meier method, and differences were analyzed using the log-rank test. Multivariable Cox proportional hazard regression was used to assess the association of TRIO scores with all-cause mortality at the latest follow-up, with hazard ratios (HRs) and 95% CI provided. Patients who received a TAVI within 30 days were excluded from the survival analysis. Variables with a *p*-value < 0.2 in the univariable regression analysis were included in multivariable models. A two-tailed *p*-value < 0.05 was considered statistically significant. All statistical analyses were performed using SPSS version 26.0 (SPSS Inc., Chicago, IL, USA).

## 3. Results

### 3.1 Baseline Characteristics

A total of 530 consecutive patients who underwent a TAVI were included in the study. The baseline characteristics are summarized in Table 1. The median age was 73 years (IQR: 68–76), and 306 patients (57.7%) were male. A total of 31 patients had chronic lung disease, and 504 patients presented with congestive heart failure. The median Society of Thoracic Surgeons (STS) score was 2.39% (IQR: 1.48–4.11%).

**Table 1.** **Baseline characteristics**.

		Total	TRIO score ≤4	TRIO score >4	*p*-value
		(n = 530)	(n = 371)	(n = 159)	
Variables					
Age (y)		73 (68–76)	72 (67–75)	75 (71–82)	<0.001
Age group					<0.001
	<70	163 (30.8)	137 (36.9)	26 (16.4)	
	70–79	292 (55.1)	216 (58.2)	76 (47.8)	
	≥80	75 (14.2)	18 (4.9)	57 (35.8)	
Male		306 (57.7)	178 (48.0)	128 (80.5)	<0.001
Body mass index, kg/m^2^		23 (20.6–24.9)	23 (20.6–25.3)	22.9 (20.4–23.7)	
Heart rate, beats/min		78 (69–86)	76 (68–84)	82 (74–94)	
Heart rate category (beats/min)					<0.001
	<90	431 (81.3)	331 (89.2)	100 (62.9)	
	≥90	99 (18.7)	40 (10.8)	59 (37.1)	
Hypertension		273 (51.5)	190 (51.2)	83 (52.20	0.835
Diabetes mellitus		118 (22.3)	86 (23.2)	32 (20.1)	0.439
Peripheral artery disease		65 (12.3)	31 (8.4)	34 (21.4)	<0.001
Chronic lung disease		31 (5.8)	0	31 (19.5)	<0.001
Coronary artery disease		180 (34.0)	119 (32.1)	61 (38.4)	0.161
Prior myocardial infarction		37 (7.0)	23 (6.2)	14 (8.8)	0.281
Prior percutaneous coronary intervention		90 (17.0)	64 (17.3)	26 (16.4)	0.801
Prior stroke		43 (8.1)	30 (8.1)	13 (8.2)	0.972
Prior pacemaker implantation		5 (0.9)	4 (1.1)	1 (0.6)	1
Atrial fibrillation		85 (16.0)	45 (12.1)	40 (25.2)	<0.001
Prior cardiac valve surgery		20 (3.8)	10 (2.7)	10 (6.3)	0.047
Congestive heart failure		504 (95.1)	345 (93.0)	159 (100)	0.001
Pulmonary artery infarction		0	0	0	
STS score, %		2.39 (1.48–4.11)	2.95 (1.65–4.43)	4.29 (2.23–7.14)	<0.001
NT-pro-BNP (pg/mL)		1955 (663–6870)	1403 (497–4258)	6150 (1831–20,207)	<0.001
TnT (pg/mL)		27.4 (16.0–67.0)	22.1 (13.6–44.6)	52 (28.5–119.1)	<0.001
Creatinine, mg/L		1.00 (0.80–1.20)	0.93 (0.76–1.12)	1.22 (0.99–2.11)	<0.001
Creatinine category (mg/L)					<0.001
	<2	485 (91.5)	368 (99.2)	117 (73.6)	
	≥2	45 (8.5)	3 (0.8)	42 (26.4)	
AST (U/L)		24 (19–31)	23 (19–28)	27 (20–46)	
AST category (U/L)					<0.001
	<40	458 (86.4)	348 (93.8)	110 (69.2)	
	≥40	72 (13.6)	23 (6.2)	49 (30.8)	
ALT (U/L)		17 (12–26)	16 (12–23)	19 (12–42)	0.001
Albumin (g/dL)		37.3 (34.3–39.9)	38 (35.3–40.1)	35.3 (32.5–38.3)	<0.001
LVEF (%)		60 (44–65)	62 (50–67)	48 (36–62)	<0.001
Mean gradient (mmHg)		54 (42–66)	56 (44–68)	50 (39–61)	<0.001
Peak velocity (m/s)		4.7 (4.2–5.2)	4.8 (4.3–5.3)	4.4 (3.9–5.0)	<0.001
Mitral regurgitation					<0.001
	None	93 (17.5)	79 (21.3)	14 (8.8)	
	Mild	229 (43.2)	177 (47.7)	52 (32.7)	
	Moderate	135 (25.5)	74 (19.9)	61 (38.4)	
	Severe	73 (13.8)	41 (11.1)	32 (20.1)	
Aortic regurgitation					0.021
	None	71 (13.4)	54 (14.6)	17 (10.7)	
	Mild	207 (39.1)	157 (42.3)	50 (31.4)	
	Moderate	148 (27.9)	93 (25.1)	55 (34.6)	
	Severe	104 (19.6)	67 (18.1)	37 (23.3)	
Tricuspid regurgitation					<0.001
	None	209 (39.4)	172 (46.4)	37 (23.3)	
	Mild	190 (35.8)	134 (36.1)	56 (35.2)	
	Moderate	87 (16.4)	54 (14.6)	33 (20.8)	
	Severe	44 (8.3)	11 (3.0)	33 (20.8)	
Pulmonary hypertension					<0.001
	None	317 (59.8)	253 (68.2)	64 (40.3)	
	Mild	101 (19.1)	63 (17.0)	38 (23.9)	
	Moderate	89 (16.8)	45 (12.1)	44 (27.7)	
	Severe	23 (4.3)	10 (2.7)	13 (8.2)	

Data are presented as the mean ± SD, median (25–75% interquartile range), and n (%). TRIO, tricuspid regurgitation impact on outcomes; STS score, Society of Thoracic Surgeons score; TnT, troponin T; NT-pro-BNP, N-terminal pro-brain natriuretic peptide; AST, aspartate aminotransferase; ALT, alanine aminotransferase; LVEF, left ventricular ejection fraction.

**Fig. 1.**
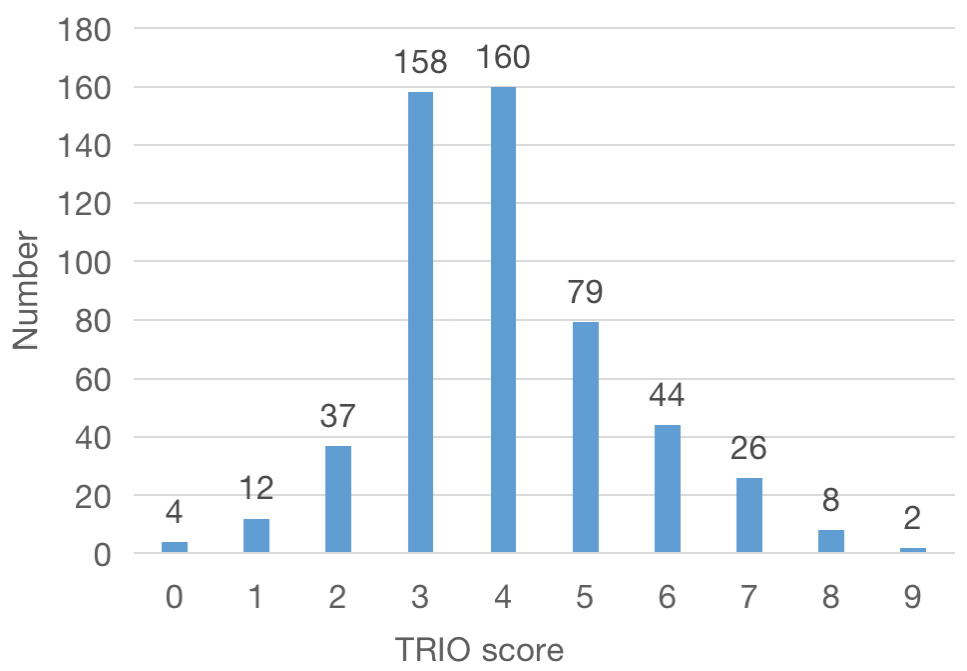
**TRIO score frequency distribution in the study cohort**. TRIO, tricuspid regurgitation impact on outcomes.

The distribution of TRIO scores is shown in Fig. 1. The median TRIO score in the cohort was 4 points, with 159 patients categorized as having a high TRIO score (>4 points) and 371 patients as having a low TRIO score (≤4 points).

The clinical and echocardiographic characteristics based on the TRIO scores are presented in Table 1. Patients with a high TRIO score were generally older and predominantly male. A higher prevalence of peripheral artery disease, chronic lung disease, atrial fibrillation, previous cardiac valve surgery, and congestive heart failure was observed in the high TRIO score group compared to the low TRIO score group. Additionally, these patients exhibited worse liver and renal functions and faster heart rates, alongside higher surgical risks.

In terms of echocardiographic findings, patients with a high TRIO score had a greater incidence of aortic regurgitation (> mild), mitral regurgitation (> mild), tricuspid regurgitation (> mild), and pulmonary hypertension (> mild) compared to the low TRIO score group. Moreover, patients with a high TRIO score had significantly decreased left ventricular ejection fractions, lower mean gradients, and reduced peak velocities.

The procedural details are shown in Table 2. Transfemoral access was the preferred approach in most procedures, and one patient received a procedure through transapical access. Pre-implantation balloon valvuloplasty was performed in 490 patients (92.5%), and post-implantation balloon valvuloplasty was performed in 220 patients (41.6%). Concomitant percutaneous coronary intervention was conducted in 52 patients (9.8%), and 47 (8.9%) underwent TAVI-in-TAVI, whereby more than one valve prosthesis was implanted during the index procedure. There were no significant differences in procedural characteristics between the high and low TRIO score groups.

**Table 2.** **Procedure characteristics**.

		Total	TRIO score ≤4	TRIO score >4	*p*-value
		(n = 530)	(n = 371)	(n = 159)	
Access					0.738
	Transfemoral	502 (94.7)	353 (95.1)	149 (93.7)	
	Trans-carotid	25 (4.7)	16 (4.3)	9 (5.7)	
	Trans-axillary	2 (0.4)	1 (0.3)	1 (0.6)	
	Transapical	1 (0.2)	1 (0.3)	0 (0)	
Pre-dilation		490 (92.5)	345 (93.0)	145 (91.2)	0.473
Post-dilation		220 (41.6)	151 (40.8)	69 (43.4)	0.580
TAVI in TAVI		47 (8.9)	28 (7.5)	19 (11.9)	0.098
Concomitant PCI		52 (9.8)	32 (8.6)	20 (12.6)	0.161

Data are presented as n (%). TAVI in TAVI, more than one valve prosthesis was implanted during the index procedure; PCI, percutaneous coronary intervention; TRIO, tricuspid regurgitation impact on outcomes; TAVI, transcatheter aortic valve implantation.

### 3.2 Clinical Outcomes

The clinical outcomes based on the TRIO scores are presented in Table 3. At 30 days, the overall mortality rate was 4.3%, the rate of stroke was 1.3%, the incidence of acute kidney injury (AKI) was 6%, pacemaker implantation was required in 6.4%, and new-onset atrial fibrillation was observed in 9.1% of the patients. No MACEs occurred in the patients who received a transapical transcatheter aortic valve replacement (TAVR) during the follow-up period. Patients with high TRIO scores had a slightly higher mortality rate compared to the low TRIO score group, although this difference was not statistically significant (6.9% vs. 3.2%, *p* = 0.056). However, patients with high TRIO scores had significantly higher rates of stroke, AKI, and MACEs compared to those with low TRIO scores (3.1% vs. 0.5%, *p* = 0.028; 10.1% vs. 4.3%, *p* = 0.011; and 11.9% vs. 4.0%, *p* = 0.001, respectively).

**Table 3.** **Clinical outcomes**.

		Total	TRIO score ≤4	TRIO score >4	*p*-value
		(n = 530)	(n = 371)	(n = 159)	
30 days outcomes					
	Mortality	23 (4.3)	12 (3.2)	11 (6.9)	0.056
	Stroke	7 (1.3)	2 (0.5)	5 (3.1)	0.028
	Acute kidney injury	32 (6.0)	16 (4.3)	16 (10.1)	0.011
	Pacemaker implantation	34 (6.4)	22 (5.9)	12 (7.5)	0.486
	New-onset atrial fibrillation	48 (9.1)	33 (8.9)	15 (9.4)	0.843
	MACEs	34 (6.4)	15 (4.0)	19 (11.9)	0.001
Latest follow-up					
	Mortality	28 (5.5)	11 (3.1)	17 (11.5)	<0.001
	MACEs	35 (6.9)	13 (3.6)	22 (14.9)	<0.001

Data are presented as n (%). MACEs, major adverse cardiovascular events: a composite of mortality, stroke, and heart failure rehospitalization; TRIO, tricuspid regurgitation impact on outcomes.

**Fig. 2.**
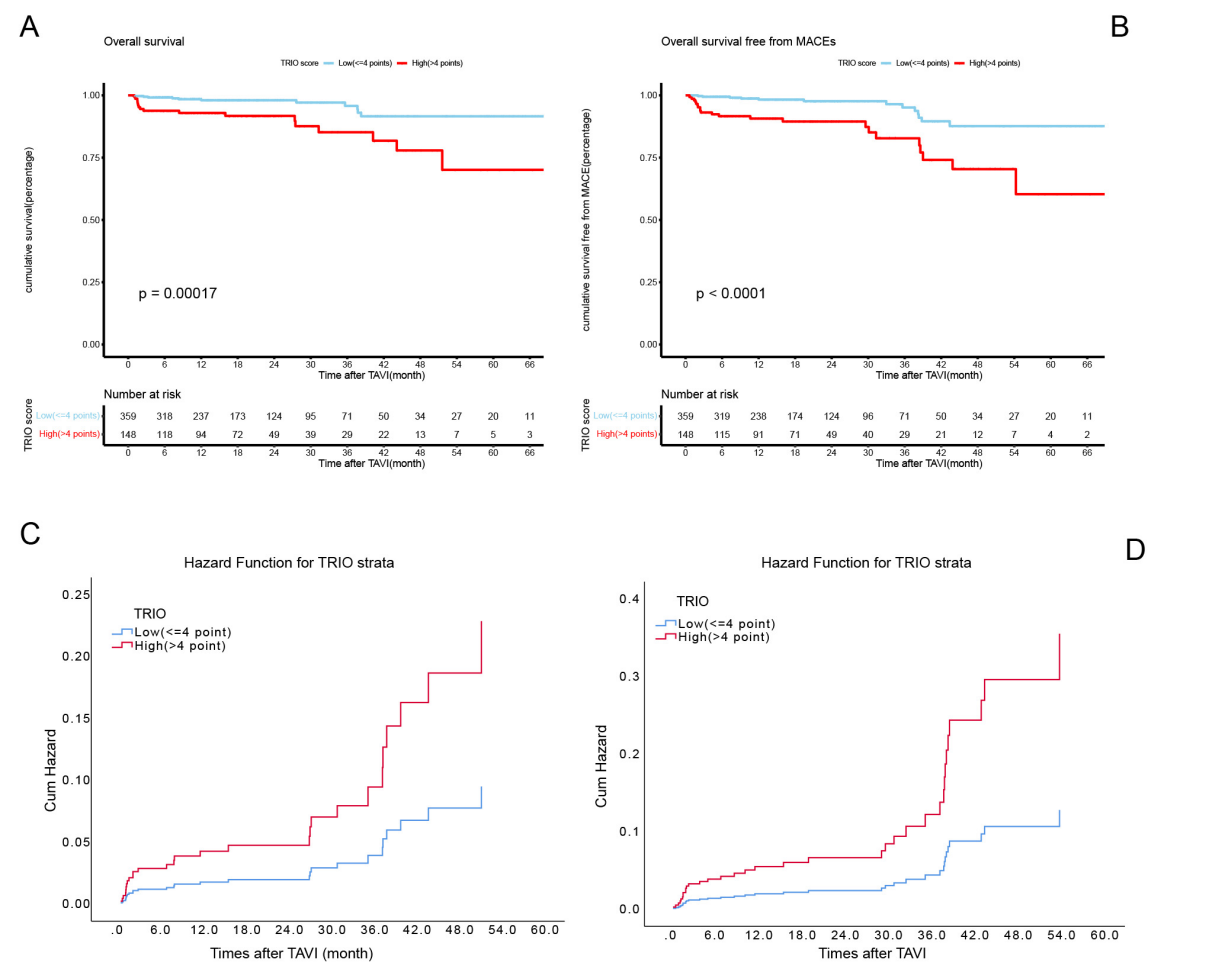
**Kaplan–Meier estimates according to low and high TRIO scores for all-cause mortality (A,C) and MACEs (B,D)**. TRIO, tricuspid regurgitation impact on outcomes; MACEs, major adverse cardiovascular events: a composite of mortality, stroke, and heart failure rehospitalization.

A total of 507 patients survived beyond 30 days after the procedure and completed at least one follow-up visit. The mean follow-up period was 22 months (95% CI: 20–23 months). Kaplan–Meier survival estimates for all-cause mortality (Fig. 2A) and MACEs (Fig. 2B) are shown. Patients with high TRIO scores had significantly higher cumulative all-cause mortality (*p* < 0.001) and MACEs (*p* < 0.001) compared to those with low TRIO scores. Crude HRs for all-cause mortality and MACEs were 3.87 (95% CI: 1.81–8.26, *p* < 0.001) and 4.25 (95% CI: 2.14–8.44, *p* < 0.001), respectively.

After adjusting for baseline confounders, the high TRIO score remained a strong independent predictor of all-cause mortality and MACEs. The adjusted HR for all-cause mortality was 3.87 (95% CI: 1.81–8.26, *p* < 0.001) (Table 4), and for MACEs, it was 2.78 (95% CI: 1.34–5.75, *p* = 0.006) (Table 5). Cumulative risk curves for all-cause mortality and MACEs are shown in Fig. 2C,D, respectively.

**Table 4.** **Univariable and multivariable analyses for all-cause mortality after a TAVI**.

		Univariable analysis		Multivariable analysis	
		HR (95% CI)	*p*-value	HR (95% CI)	*p*-value
Age		1.05 (0.99–1.10)	0.144	-	-
Male sex		1.74 (0.79–3.85)	0.171	-	-
Body mass index, kg/m^2^		0.98 (0.89–1.09)	0.723		
Heart rate ≥90 beats/min		1.74 (0.77–3.95)	0.187	-	-
Hypertension		0.79 (0.38–1.67)	0.534	-	-
Diabetes mellitus		0.94 (0.38–2.33)	0.898	-	-
Peripheral artery disease		2.00 (0.85–4.70)	0.115	-	-
Chronic lung disease		1.76 (0.53–5.83)	0.358		
Coronary artery disease		0.82 (0.38–1.80)	0.623	-	-
STS score		1.11 (1.07–1.15)	<0.001	1.10 (1.05–1.15)	<0.001
Creatinine, mg/L		1.32 (1.19–1.47)	<0.001	1.24 (1.09–1.41)	0.001
Creatinine ≥2, mg/L		6.31 (2.83–14.10)	<0.001		
Albumin, g/dL		0.90 (0.84–0.98)	0.013	-	-
AST ≥40 U/L		1.77 (0.72–4.36)	0.217		
LVEF (%)		0.98 (0.96–0.99)	0.033	-	-
Mitral regurgitation					
	None	Ref	-		
	Mild	0.81 (0.24–2.70)	0.734		
	Moderate	1.00 (0.28–3.57)	0.992		
	Severe	3.26 (1.02–10.34)	0.046		
Aortic regurgitation					
	None	Ref	-		
	Mild	1.64 (0.36–7.51)	0.521		
	Moderate	2.04 (0.43–9.63)	0.366		
	Severe	2.61 (0.55–12.31)	0.225		
Tricuspid regurgitation				-	-
	None	Ref	-		
	Mild	2.51 (0.94–6.70)	0.066		
	Moderate	3.22 (1.08–0.58)	0.036		
	Severe	2.52 (0.63–10.07)	0.192		
Pulmonary hypertension					
	None	Ref	-		
	Mild	1.96 (0.80–4.81)	0.140		
	Moderate	2.05 (0.81–5.22)	0.131		
	Severe	1.48 (0.19–11.45)	0.705		
TRIO score (per 1 point increase)		1.56 (1.25–1.93)	<0.001		
TRIO score (point)					
	≤4	Ref	-	Ref	-
	>4	3.87 (1.81–8.26)	<0.001	2.41 (1.08–5.37)	0.032

HR, hazard ratio; 95% CI, 95% confidence interval; STS score, Society of Thoracic Surgeons score; AST, aspartate aminotransferase; LVEF, left ventricular ejection fraction; TRIO, tricuspid regurgitation impact on outcomes; TAVI, transcatheter aortic valve implantation; Ref, reference.

**Table 5.** **Univariable and multivariable analyses for MACEs after a TAVI**.

		Univariable analysis		Multivariable analysis	
		HR (95% CI)	*p*-value	HR (95% CI)	*p*-value
Age		1.05 (1.00–1.10)	0.049	-	-
Male sex		1.83 (0.90–3.75)	0.097	-	-
Body mass index, kg/m^2^		0.99 (0.90–1.08)	0.766		
Heart rate ≥90 beats/min		2.01 (0.98–4.11)	0.056	-	-
Hypertension		0.98 (0.51–1.90)	0.952		
Diabetes mellitus		1.42 (0.68–2.96)	0.348		
Peripheral artery disease		1.70 (0.77–3.76)	0.187	-	-
Chronic lung disease		2.03 (0.72–5.75)	0.184	-	-
Coronary artery disease		0.83 (0.42–1.67)	0.602		
STS score		1.11 (1.07–1.15)	<0.001	1.09 (1.05–1.15)	0.002
Creatinine, mg/L		1.32 (1.19–1.45)	<0.001	1.21 (1.07–1.37)	<0.001
Creatinine ≥2, mg/L		6.22 (3.03–12.79)	<0.001		
Albumin, g/dL		0.92 (0.85–0.99)	0.026	-	-
AST ≥40 U/L		1.40 (0.58–3.37)	0.457		
LVEF (%)		0.98 (0.96–1.01)	0.075	-	-
Mitral regurgitation					
	None	Ref	-		
	Mild	0.72 (0.27–1.95)	0.522		
	Moderate	0.88 (0.31–2.55)	0.820		
	Severe	2.07 (0.75–5.72)	0.158		
Aortic regurgitation					
	None	Ref	-		
	Mild	0.59 (0.22–1.59)	0.293		
	Moderate	0.87 (0.32–2.38)	0.866		
	Severe	0.86 (0.30–2.47)	0.856		
Tricuspid regurgitation					
	None	Ref	-		
	Mild	1.61 (0.70–3.67)	0.261		
	Moderate	1.91 (0.73–5.01)	0.191		
	Severe	2.35 (0.80–6.87)	0.120		
Pulmonary hypertension					
	None	Ref	-		
	Mild	1.51 (0.67–3.40)	0.317		
	Moderate	1.58 (0.68–3.67)	0.286		
	Severe	1.06 (0.14–7.99)	0.955		
TRIO score (per 1 point increase)		1.62 (1.34–1.97)	<0.001		
TRIO score (point)					
	≤4	Ref	-	Ref	-
	>4	4.25 (2.14–8.44)	<0.001	2.78 (1.34–5.75)	0.006

HR, hazard ratio; 95% CI, 95% confidence interval; STS score, Society of Thoracic Surgeons score; AST, aspartate aminotransferase; LVEF, left ventricular ejection fraction; TRIO, tricuspid regurgitation impact on outcomes; TAVI, transcatheter aortic valve implantation; MACEs, major adverse cardiovascular events; Ref, reference.

**Fig. 3.**
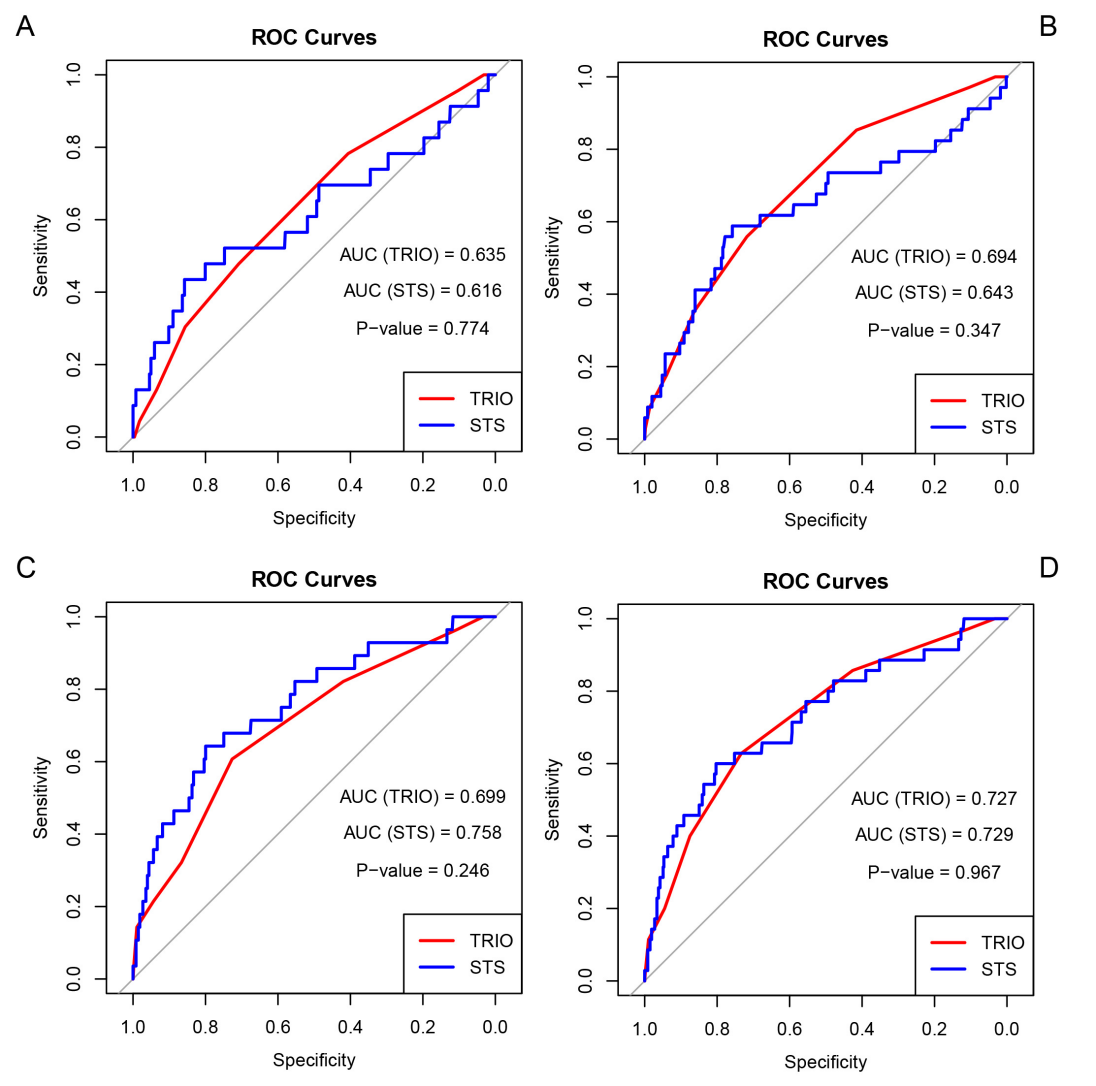
**The receiver operating characteristic curve of TRIO and STS score predicts mortality and MACEs**. (A) The 30-day mortality. (B) The 30-day rate of MACEs. (C) Mortality after 30 days. (D) MACEs after 30 days. TRIO, tricuspid regurgitation impact on outcomes; ROC, receiver operating characteristic; AUC, area under the curve; STS, Society of Thoracic Surgeons; MACEs, major adverse cardiovascular events: a composite of mortality, stroke, and heart failure rehospitalization.

The TRIO and STS scores demonstrated excellent discrimination in predicting mortality and MACEs. Furthermore, the effects of these scores on predicting mortality and MACEs were comparable (Fig. 3). Although the STS score had a slightly higher area under the curve (AUC) in predicting 30-day mortality, no statistical differences were observed (TRIO vs. STS = 0.699 vs. 0.758, *p* = 0.246).

## 4. Discussion

This single-center retrospective study evaluated the prognostic impact of the TRIO scores in 530 patients who underwent a TAVI. The main findings of our study were as follows: (1) the rates of all-cause mortality and MACEs were significantly higher in patients with high TRIO scores. (2) the TRIO score was independently associated with all-cause mortality and MACEs in patients who had undergone a TAVI.

To our knowledge, this was the first study to assess the prognostic value of the TRIO score in patients who had received a TAVI. Predictors of poor outcomes following a TAVI included severe lung disease, chronic kidney disease, frailty, left ventricular dysfunction, pulmonary hypertension, and severe mitral regurgitation [10]. Capodanno *et al*. [11] developed the OBSERVANT risk score, which includes seven factors: renal dysfunction, pre-operative state, New York Heart Association (NYHA) functional class, pulmonary artery hypertension, diabetes, previous balloon aortic valvuloplasty, and left ventricular ejection fraction. These factors are readily available in clinical practice; however, the OBSERVANT score specifically predicts 30-day mortality rather than long-term outcomes. In a retrospective analysis of 530 patients, Hermiller *et al*. [12] devised a risk score based on albumin levels, Charlson comorbidity index, home oxygen use, and STS score to predict both early and late mortality risk after a TAVI. While this score incorporates frailty and nutritional status, essential aspects of patient prognosis, such information may not always be routinely collected. Additionally, reliance on the STS score, which can be time-consuming, limits the widespread applicability of the Hermiller *et al*. [12] devised score in everyday practice.

Neither of these risk scores incorporates parameters of right ventricular function, liver function, or lung function, which are increasingly recognized as crucial predictors of outcomes in patients following a TAVI. The TRIO score was originally developed and validated to predict mortality risk in patients with tricuspid regurgitation and pulmonary hypertension [8, 13]. Since many patients undergoing a TAVI also present with concomitant tricuspid regurgitation and pulmonary hypertension, conditions strongly linked to increased mortality risk [14, 15, 16], we sought to extend these findings to a TAVI cohort. Our results demonstrated that the TRIO score exhibited excellent discrimination for predicting all-cause mortality, independent of tricuspid regurgitation or pulmonary hypertension status. We also compared the effect of the TRIO score to the STS score in predicting all-cause mortality and MACEs and observed no difference. However, the TRIO score was more convenient and concise than the STS score, while the STS score was more time-consuming. Our study established its prognostic value and highlighted the significant association between a high TRIO score and worse clinical outcomes. The TRIO score remained a predictor of all-cause mortality after adjusting for confounding factors, including comorbidities, renal function, and cardiac function.

Right ventricular dysfunction (RVD) is considered a late marker of left-sided heart disease and is reported in approximately 25% of AS patients [17, 18, 19]. Several studies have investigated the prognostic impact of RVD in AS patients, yielding conflicting results [20, 21, 22]. Echocardiographic assessments of right ventricular function typically include tricuspid annular plane systolic excursion, the S’ wave, right ventricular size, and fractional area change. Although the TRIO score does not explicitly incorporate these parameters, it does include tricuspid regurgitation, which serves as a surrogate marker for the progression of RVD and right-sided heart failure. Significant tricuspid regurgitation has been associated with poorer outcomes, particularly in patients with significant tricuspid valve dysfunction [8]. RVD and tricuspid regurgitation contribute to chronic systemic venous congestion, which can lead to end-organ damage, including liver dysfunction and elevated serum aspartate transaminase levels [23]. Thus, the TRIO score partially reflects right-sided heart function and has been shown to predict a worse prognosis for patients after a TAVI.

Our study also demonstrated a correlation between a higher TRIO score and an increased risk of stroke within 30 days following a TAVI. Stroke is a particularly concerning complication of the TAVI, and the TRIO score may help identify patients at an elevated risk who could benefit from cerebral embolic protection devices. These devices have shown promise in reducing stroke incidence [24]; however, a recent randomized controlled trial reported no significant effect on the overall incidence of periprocedural stroke [25]. Therefore, careful patient selection is critical, and the TRIO score may provide a valuable reference for determining appropriate candidates for embolic protection devices.

AKI is another common complication following a TAVI, with reported prevalence rates ranging from 3.4% to 57%, primarily in older people and high-risk populations [26]. Moreover, the development of AKI after a TAVI is associated with increased short- and long-term mortality [27, 28]. In our study, patients with a higher TRIO score were more likely to develop AKI following a TAVI, which could be attributed to several factors included in the TRIO score, such as advanced age, baseline renal dysfunction, lung disease, and heart failure—all of which have been previously identified as risk factors for AKI after a TAVI [26, 29]. Consequently, patients with elevated TRIO scores may benefit from targeted medical interventions to optimize heart failure management and lung function prior to a TAVI.

## 5. Limitations

Several limitations of this study must be acknowledged. First, this was a retrospective analysis of patients from a single center, which may limit the generalizability of our findings. However, the study reflected the “real-world” daily TAVI practices. Second, the sample size of our cohort was relatively small, and the results should be validated in larger, multicenter cohorts.

## 6. Conclusions

In conclusion, our study demonstrated that a higher TRIO score is significantly associated with an increased risk of all-cause mortality and MACEs in aortic stenosis patients after a TAVI. These results propose that the TRIO score may serve as a simple and effective prognostic marker in patients after a TAVI. Thus, advanced therapeutic interventions for patients with high TRIO scores could improve clinical outcomes.

## Availability of Data and Materials

The datasets used and/or analyzed during the current study are available from the corresponding author on reasonable request.
